# Triple negative breast cancer development can be selectively suppressed by sustaining an elevated level of cellular cyclic AMP through simultaneously blocking its efflux and decomposition

**DOI:** 10.18632/oncotarget.13601

**Published:** 2016-11-25

**Authors:** Wei Wang, Yue Li, Jessica Y. Zhu, Dongdong Fang, Han-Fei Ding, Zheng Dong, Qing Jing, Shi-Bing Su, Shuang Huang

**Affiliations:** ^1^ Research Center for Traditional Chinese Medicine Complexity System, Shanghai University of Traditional Chinese Medicine, Shanghai, China; ^2^ Department of Anatomy and Cell Biology, University of Florida College of Medicine, Gainesville, FL, USA; ^3^ Georgia Cancer Center, Augusta University, Augusta, GA, USA; ^4^ Department of Anatomy and Cell Biology, Medical College of Georgia, Augusta University, Augusta, GA, USA; ^5^ Changhai Hospital, Shanghai, China; ^6^ E-institute of Shanghai Municipal Education Committee, Shanghai University of Traditional Chinese Medicine, Shanghai, China

**Keywords:** cAMP, MRP, PDE, TNBC, cell growth

## Abstract

Triple negative breast cancer (TNBC) has the highest mortality among all breast cancer types and lack of targeted therapy is a key factor contributing to its high mortality rate. In this study, we show that 8-bromo-cAMP, a cyclic adenosine monophosphate (cAMP) analog at high concentration (> 1 mM) selectively suppresses TNBC cell growth. However, commonly-used cAMP-elevating agents such as adenylyl cyclase activator forskolin and pan phosphodiesterase inhibitor 3-isobutyl-1-methylxanthine (IBMX) are ineffective. Inability of cAMP elevating agents to inhibit TNBC cell growth is due to rapid diminution of cellular cAMP through efflux and decomposition. By performing bioinformatics analyses with publically available gene expression datasets from breast cancer patients/established breast cancer cell lines and further validating using specific inhibitors/siRNAs, we reveal that multidrug resistance-associated protein 1/4 (MRP1/4) mediate rapid cAMP efflux while members PDE4 subfamily facilitate cAMP decomposition. When cAMP clearance is prevented by specific inhibitors, forskolin blocks TNBC's *in vitro* cell growth by arresting cell cycle at G1/S phase. Importantly, cocktail of forskolin, MRP inhibitor probenecid and PDE4 inhibitor rolipram suppresses TNBC *in vivo* tumor development. This study suggests that a TNBC-targeted therapeutic strategy can be developed by sustaining an elevated level of cAMP through simultaneously blocking its efflux and decomposition.

## INTRODUCTION

Triple-negative breast cancer (TNBC), a subset of breast cancer with the absence of estrogen and progesterone receptors (ERs and PRs) and lack of amplification of the human epidermal growth factor receptor 2 (HER2) gene, accounts for 15–20% of all breast cancer [1, 2]. Compared to hormone receptor-positive breast cancer, TNBC more commonly affects younger patients, has a higher prevalence in African-American women and often follows an aggressive clinical course including a high incidence of visceral and brain metastases [3, 4]. Although cytotoxic chemotherapy, the only approved therapy for TNBC, leads to an initial significant response rate, patients frequently suffer from relapses and subsequent mortality [5]. Therefore, innovative therapeutic strategies targeting TNBC are urgently needed [6].

Agents that increase cellular cyclic AMP (cAMP) level have been found to suppress cell growth in various cell types by triggering apoptosis or cell-cycle arrest [7–9]. These agents include cAMP analogs [dibutyryl-cAMP, 8-bromo-cAMP (8-Br-cAMP) and 8-chloro-cAMP], adenylate cyclase activators and phosphodiesterase (PDE) inhibitors. Unfortunately, none of them has been recommended for cancer therapy because they display high toxicity at the dose that can effectively inhibit *in vitro* tumor cell growth or *in vivo* tumor development [10]. We reason that understanding the cause that these agents elicit anti-tumor effect only at very high doses can help developing strategies in which cAMP-elevating agents can be utilized at reduced and nontoxic doses.

Cellular events led by cAMP are generally mediated through protein kinase A (PKA) and cAMP-regulated guanine nucleotide exchange factors [11]. PKA-II is preferentially expressed in normal non-proliferating tissues and growth-arrested cells whereas PKA-I is overexpressed in cancer cells [12]. Since cAMP analogs inhibit PKA-I expression while they promote the formation of PKA-II in cancer cells, the differential regulation of PKA isozymes by cAMP may be one of the explanations for cAMP's growth-suppressive activity [13, 14]. Recent evidences also show that cAMP can suppress cell growth by interfering with c-Raf-MEK1/2-Erk signaling pathway [15, 16], attenuating the expression of anti-apoptotic protein Bcl_2_ [17] or inducing the expression of cell-cycle inhibitor p27kip1 [18]. Moreover, cAMP can stimulate cell differentiation [13, 19] and mesenchymal-to-epithelial transition [20], which may also lead to cell growth inhibition.

In this study, we show that 8-Br-cAMP at concentration > 1 mM inhibits growth of TNBC but not ER+ cells. Surprisingly, TNBC cell growth was little affected by adenylate cyclase activator forskolin and pan-PDE inhibitor 3-isobutyl-1-methyl-xanthene (IBMX). To elucidate this apparent discrepancy, we uncover that the inability of forskolin/IBMX to inhibit TNBC cell growth is due to a rapid diminution of cellular cAMP by multidrug resistance-associated protein (MRP)-mediated efflux. With the aid of short interfering RNAs (siRNAs), MRP1 and MRP4 are identified as the members of MRP family facilitating rapid cAMP efflux in TNBC cells. Meanwhile, we provide evidences that multiple PDE4 isotypes can diminish cellular cAMP when MRPs are blocked. Finally, we demonstrate that cocktail of forskolin, probenecid (pan-MRP inhibitor) and rolipram (PDE4 inhibitor) effectively inhibit *in vitro* cell growth and *in vivo* tumor development of TNBC cells.

## RESULTS

### High concentration of cAMP analog but not cAMP-elevating agents inhibits TNBC cell growth

**Figure 1:**
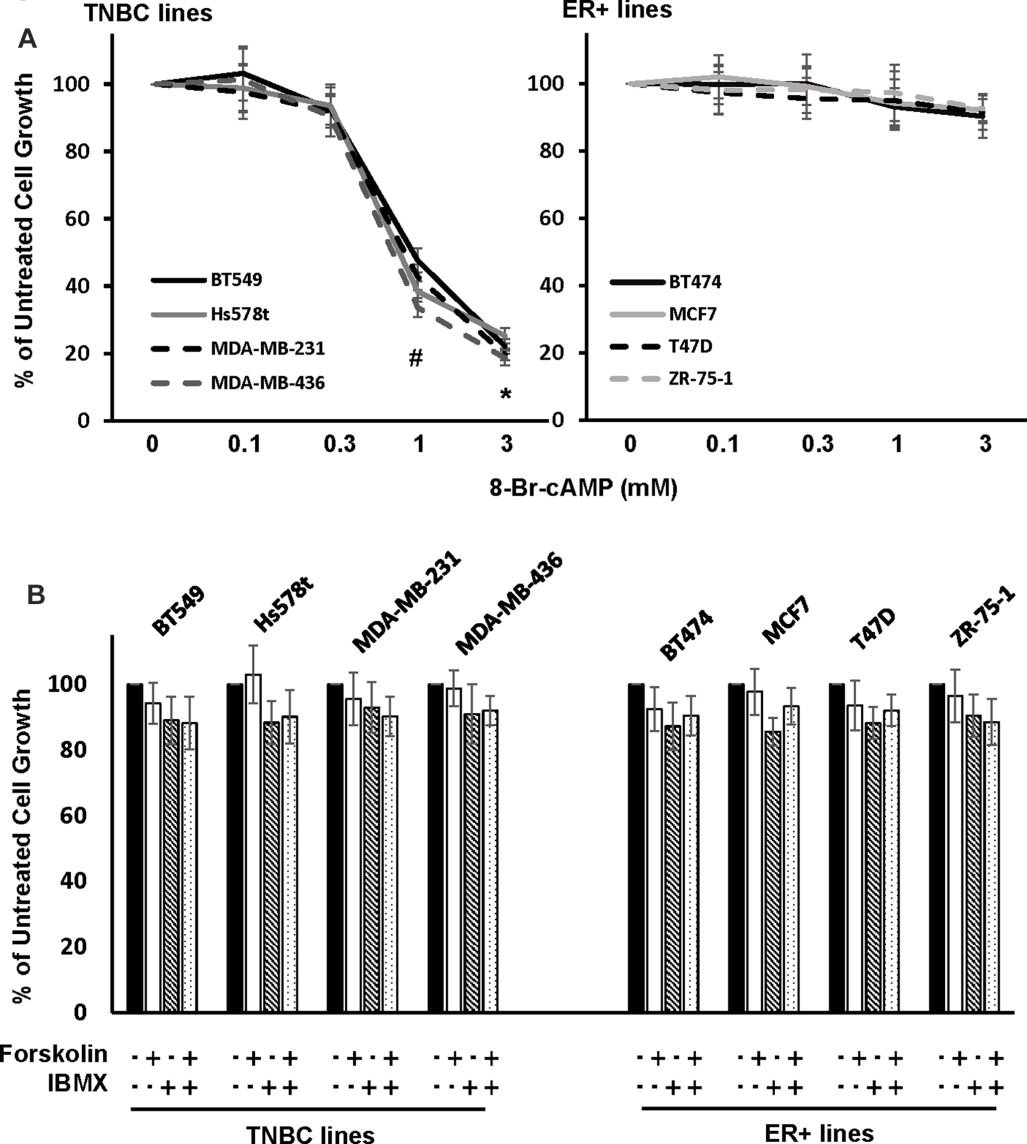
Effect of cAMP-elevating agents on TNBC and ER+ breast cancer cell growth (**A, B**) TNBC (A) or ER+ breast cancer cells (B) were treated with various concentration of 8-Br-cAMP for 4 days followed by MTT assay to determine cell growth. Data are means ± SD (*n* = 4). *P < 0.005 *vs* control. (**C**) TNBC and ER+ breast cancer cells were treated with 10μM forskolin in the absence or presence of 100 μM IBMX for 4 days followed by MTT assay to assess cell growth. Data are means ± SD (*n* = 4).

A recent study reported that various cAMP-elevating agents were able to inhibit growth of MDA-MB-231, a TNBC line [21]. To determine if the same could be generalized to other breast cancer cell lines, we examined the effect of 8-Br-cAMP, a PDE-resistant cAMP analog, on growth of 4 TNBC and 4 ER+ cell lines. MTT assay showed that 8-Br-cAMP at concentration > 1 mM inhibited growth of TNBC but not ER+ lines (Figure 1A). Further clonogenic assay showed that 1 mM 8-Br-cAMP reduced more than 75% of colonies formed in MDA-MB-231 cells while only 15% reduction the number of in formed colonies was detected in MCF7 cells (Supplementary Figure S1). These results suggest that TNBC cells are selectively sensitive to elevated level of cellular cAMP.

The necessity of 8-Br-cAMP to inhibit TNBC cell growth at concentration > 1mM led us to investigate whether cAMP-elevating agents would be more effective. We treated TNBC cells with forskolin, an adenylyl cyclase activator, and IBMX, a pan-PDE inhibitor alone or together for 4 days followed by cell growth analysis. MTT assay showed that growth of neither TNBC nor ER+ cells was significantly altered by forskolin and IBMX alone or together (Figure 1B).

### Cellular cAMP is rapidly diminished in TNBC cells through efflux

**Figure 2:**
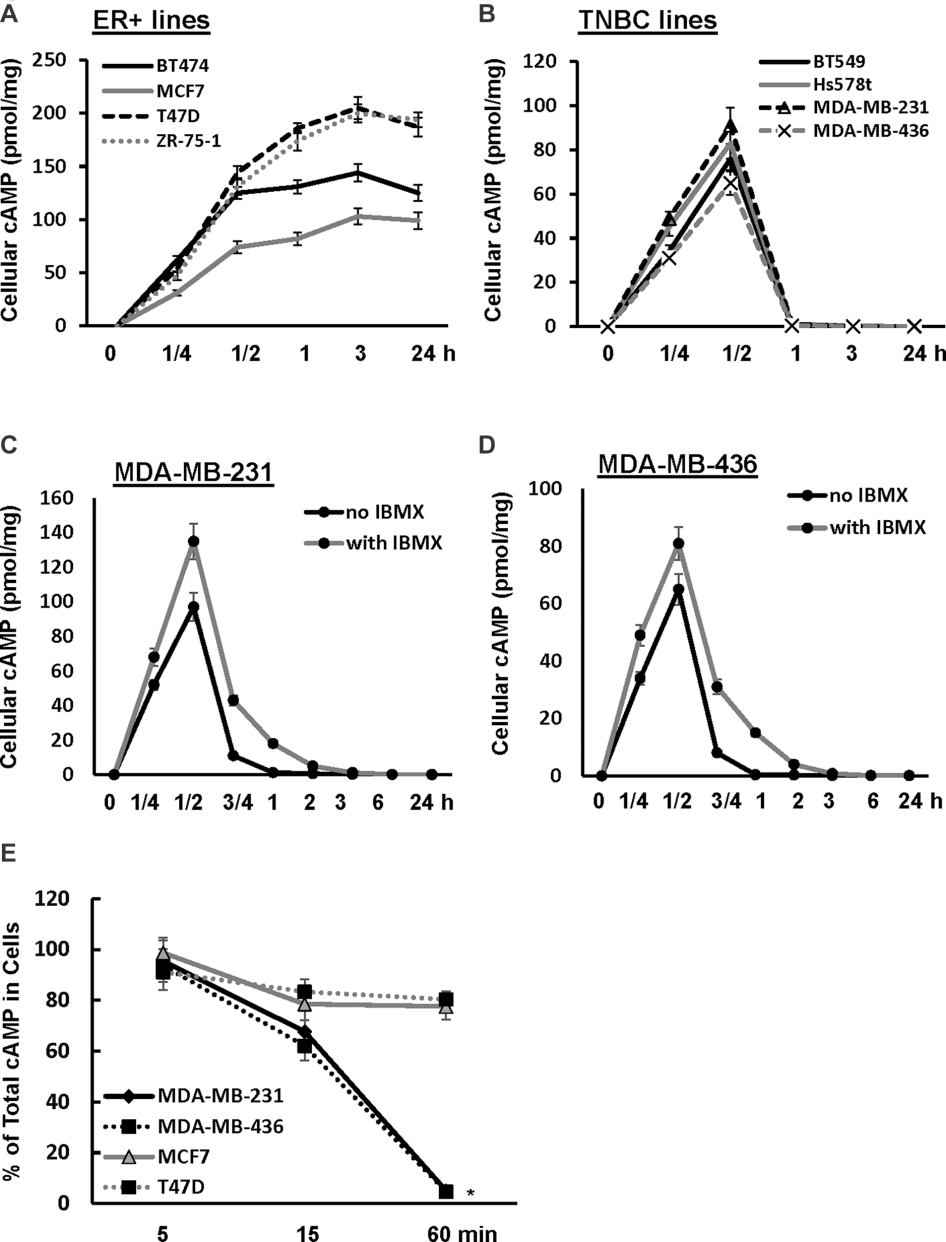
Cellular cAMP is rapidly diminished in TNBC cells (**A, B**) TNBC (A) and ER+ breast cancer cells (B) were treated with 10μM forskolin for 1/4, 1/2, 1, 3 and 24 h followed by assessing the amount of cellular cAMP. Data are means ± SD (*n* = 3). (**C, D**) MDA-MB-231 (C) or MDA-MB-436 cells (D) were treated with 10 μM forskolin in the absence or presence of 100μM IBMX for 0, 1/4, 1/2, 3/4, 1, 2, 3, 6 and 24 h, then collected and analyzed for the amount of cellular cAMP. Data are means ± SD (*n* = 3). (**E**) MDA-MB-231, MDA-MB-436, MCF-7 and T47D cells were treated with 10μM forskolin and 100 μM IBMX for 5, 15 and 60 min. Both cells and media were collected and analyzed for amount of cellular and secreted cAMP. Total cAMP = cellular cAMP + secreted cAMP. Data are means ± SD (*n* = 3). *P < 0.01 *vs* 0 min.

The discrepancy on the effect of TNBC cell growth between high concentration of 8-Br-cAMP and cAMP-elevating agents indicated the possibility that forskolin/IBMX was unable to elevate cellular cAMP to a level sufficient to inhibit TNBC cell growth. To test it, we examined the effect of forskolin on cellular cAMP concentration in both TNBC and ER+ lines. In ER+ cells (BT474, MCF7, T47D and ZR-75-1), forskolin induced a fast and sustained elevation of cellular cAMP in the entire 24-h stimulation period (Figure 2A). In contrast, forskolin transiently increased the level of cellular cAMP which was rapidly diminished after 30 min in TNBC cells (BT549, Hs578T, MDA-MB-231 and MDA-MB-436) and nearly subsided to the level of prior treatment at 1 h (Figure 2B). These results demonstrate that cellular cAMP is quite stable in ER+ cells whereas it is quickly cleared in TNBC cells. To determine the role of PDEs in rapid diminution of cellular cAMP, MDA-MB-231 and MDA-MB-436 cells were pretreated with IBMX prior to forskolin stimulation. Analysis of cAMP in cells showed that IBMX was unable to prevent the clearance of cAMP although the rate was moderately slower (Figure 2C and 2D).

To determine whether efflux is responsible for rapid clearance of cellular cAMP in TNBC cells, we determined what percentage of total cAMP was cellular in MDA-MB-231 and MDA-MB-436 cells at various times of forskolin/IBMX stimulation. Over 90% of total cAMP was detected in cells at 5-min of stimulation and total cAMP in cells reduced to approximately 60–70% at 15 min (Figure 2E). At 1 h, only less than 5% of total cAMP remained in cells (Figure 2E). In parallel, we determined how cAMP was distributed in ER+ MCF7 and T47D cells upon forskolin/IBMX treatment. Similar to TNBC cells, we detected that over 90% of total cAMP was cellular at 5 min but percentage of total cAMP in cells decreased to approximately 80% at 15 min (Figure 2E). Contrary to TNBC cells, there was no further reduction in the percentage of total cAMP that was cellular in both MCF7 and T47D cells (Figure 2E). These results indicate that rapid diminution of cellular cAMP is unique to TNBC cells and is mainly mediated by efflux.

### MRP1 and MRP4 facilitate cAMP efflux in TNBC cells

**Figure 3:**
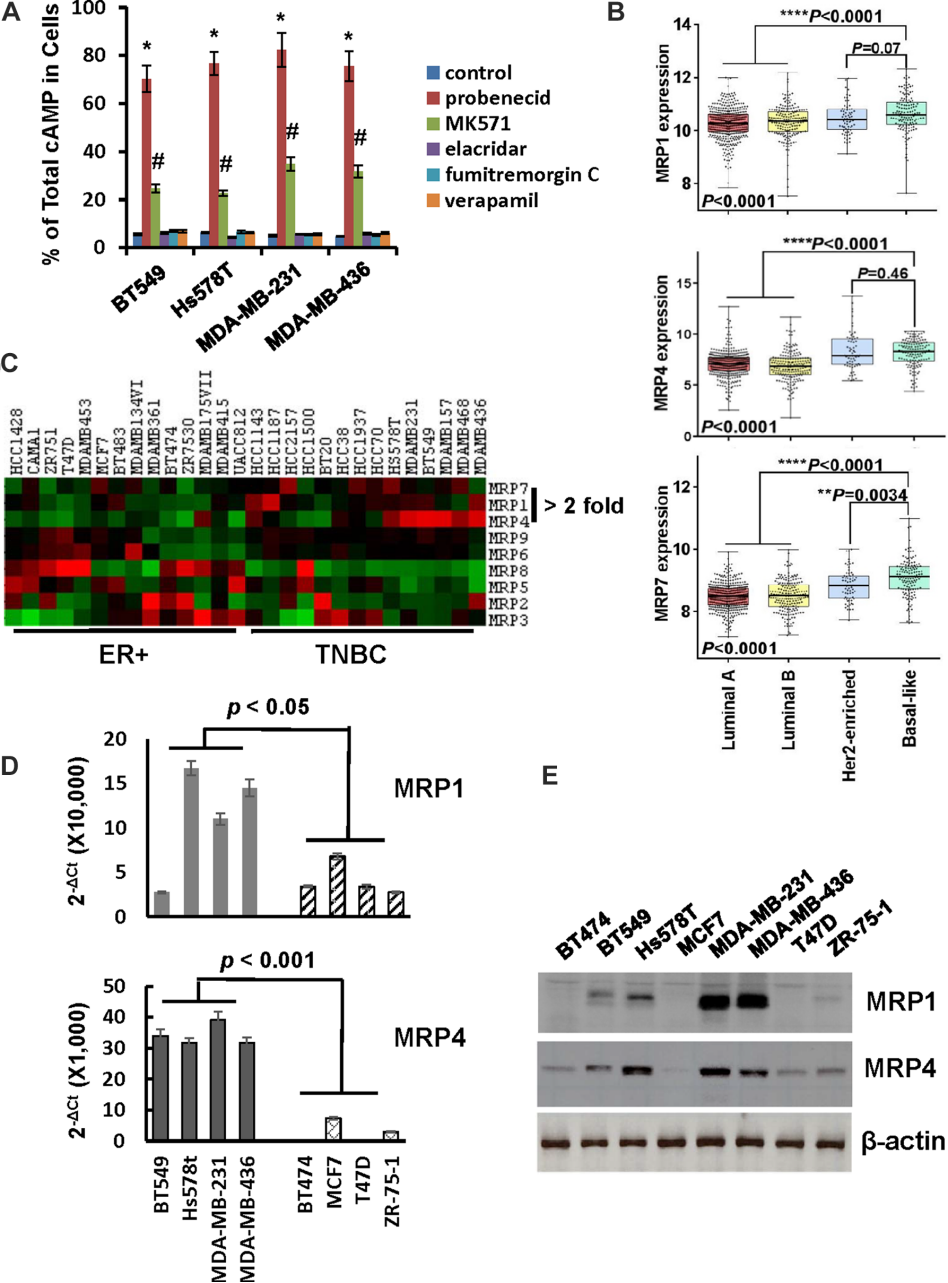
Expression of MRP1 and MRP4 is elevated in TNBC cells (**A**) MDA-MB-231 and MDA-MB-436 cells were pretreated with 1 mM probenecid, 50 μM MK571, 0.5 μM elacridar hydrochloride, 10μM fumitremorgin C or 100 μM verapamil for 1 h and then incubated with 10 μM forskolin and 100 μM IBMX for 1 h. Cells and media were collected and analyzed for levels of cellular and secreted cAMP respectively. Data are means ± SD (*n* = 3). *P < 0.001 *vs* control; #*P* < 0.01 *vs* control. (**B**) Box-and-whisker plot was generate to show the expression of MRP1, MRP4 and MRP7 in breast tumor subtypes. (**C**) Heat map was generated to reveal the expression of MRP family members in established ER+ and TNBC cell lines. (**D**) QRT-PCR of MRP1 and MRP4 mRNA in breast cancer cell lines. Level of β-actin mRNA was used as an internal control for standardization. Data are means ± SD (*n* = 3). (**E**) Overnight-cultured cells were lysed and cell lysates were subjected to western blotting to detect MRP1, MRP4 and β-actin with the respective antibodies.

Members of MRP family are efficient cAMP efflux pumps [22]. To investigate the potential role of MRPs in cAMP efflux in TNBC cells, BT549, Hs578t, MDA-MB-231 and MDA-MB-436 cells were pretreated with pan MRP inhibitor probenecid, MPR1-selective inhibitor MK571, MDR inhibitor elacridar hydrochloride, BCRP inhibitor fumitremorgin C or verapamil followed by 1-h of forskolin/IBMX stimulation. More than 70% and approximately 20–35% of total cAMP was found to be cellular in probenecid- and MK571-pretreated cells respectively (Figure 3A). In contrast, none of the other inhibitors increased the percentage of total cAMP over the control (Figure 3A). These results support the notion that MRPs are responsible for cAMP efflux.

To identify the particular MRP family members that facilitate cAMP efflux in TNBC cells, we initially compared the expression of MRP family members in luminal-A, luminal-B, HER2-enriched and basal-like breast tumors using publicly available TCGA dataset. Levels of MRP1, MRP4 and MRP7 mRNA, but not other members of MRP family are significantly higher in basal-like breast tumors compared to those in luminal-A/B breast tumors (Figure 3B and Supplementary S2). Since the majority of TNBC are of basal-like phenotype and the majority of tumors expressing ‘basal’ markers are TNBCs [23], expression of MRP1, MRP4 and MRP7 is most likely greater in TNBCs. Subsequently, we analyzed MRP expression profile on established breast cancer cell lines using datasets available from Cancer Cell Line Encyclopedia. Consistent with results from human breast tumor tissues, MRP1, MRP4 and MRP7 were expressed at least 2-fold higher in TNBC lines than ER+ ones (Figure 3C). To validate the findings derived from public datasets, we examined the expression of MRP family members in a panel of breast cancer cell lines. QRT-PCR showed that MRP2, MRP6, MRP8 and MRP9 were hardly detectable in all lines while MRP3 and MRP7 were similarly expressed in ER+ and TNBC lines and level of MRP5 mRNA was higher in ER+ lines (Supplementary Figure S3). In contrast, expression of MRP1 and MRP4 was much greater in TNBC lines than ER+ ones (Figure 3D). Similarly, western blot also showed that levels of MRP1 and MRP4 were higher in TNBC lines than ER+ ones (Figure 3E).

**Figure 4:**
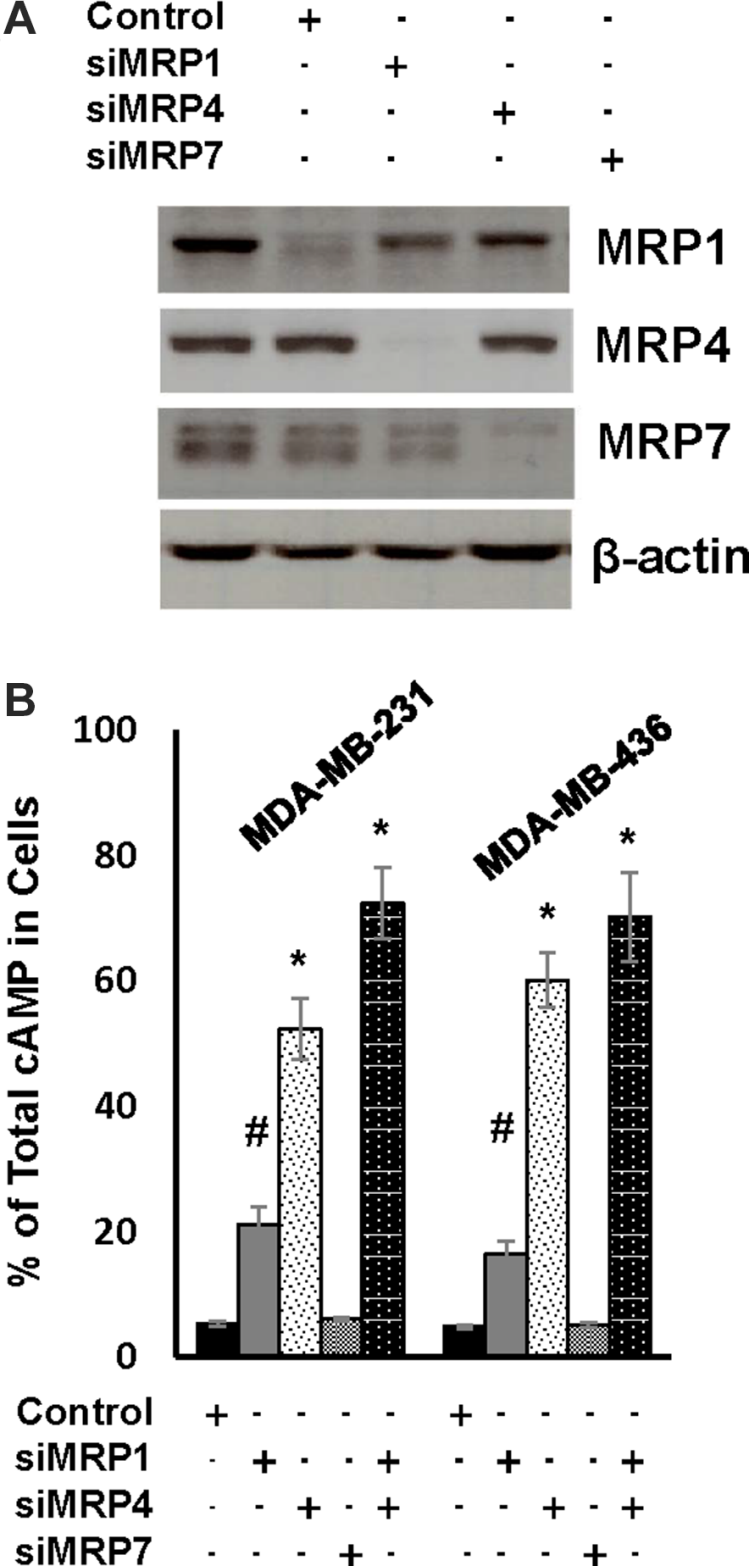
MRP1 and MRP4 facilitate rapid cAMP diminution in TNBC cells (**A**) MDA-MB-231 cells were transfected with control siRNA, MRP1, MRP4 or MRP7 siRNA pool for 4 days and then lysed for western blotting to detect MRP1, MRP4, MRP7 or β-actin with the respective antibodies. (**B**) MDA-MB-231 and MDA-MB-436 cells were transfected with control siRNA, MRP1, MRP4 or MRP7 siRNA pool for 4 days followed by 1-h treatment of 10 μM forskolin and 100 μM IBMX. Cells and media were collected and analyzed for the amount of cellular and secreted cAMP respectively. Data are means ± SD (*n* = 3). *P < 0.01 *vs* control siRNA.

We next investigated the effect of depleting MRP1, MRP4 or MRP7 on cAMP efflux by introducing their respective siRNA pools into MDA-MB-231 and MDA-MB-436 cells (Figure 4A). Knockdown of MRP1 rendered 21 and 16% of total cAMP cellular in MDA-MB-231 and MDA-MB-436 cells respectively while knockdown of MRP4 kept 52 and 60% of total cAMP in cells respectively (Figure 4B). Contrarily, knockdown of MRP7 displayed little effect on the level of cellular cAMP (Figure 4B). When both MRP1 and MRP4 were silenced together, over 70% of total cAMP wwas found to be cellular in these two lines (Figure 4B). These results suggest that MRP4 is the principal cAMP efflux pump in TNBC cells whereas MRP1 also contributes to this event.

### PDE4-mediated decomposition plays a role in clearance of cellular cAMP in TNBC cells

**Figure 5:**
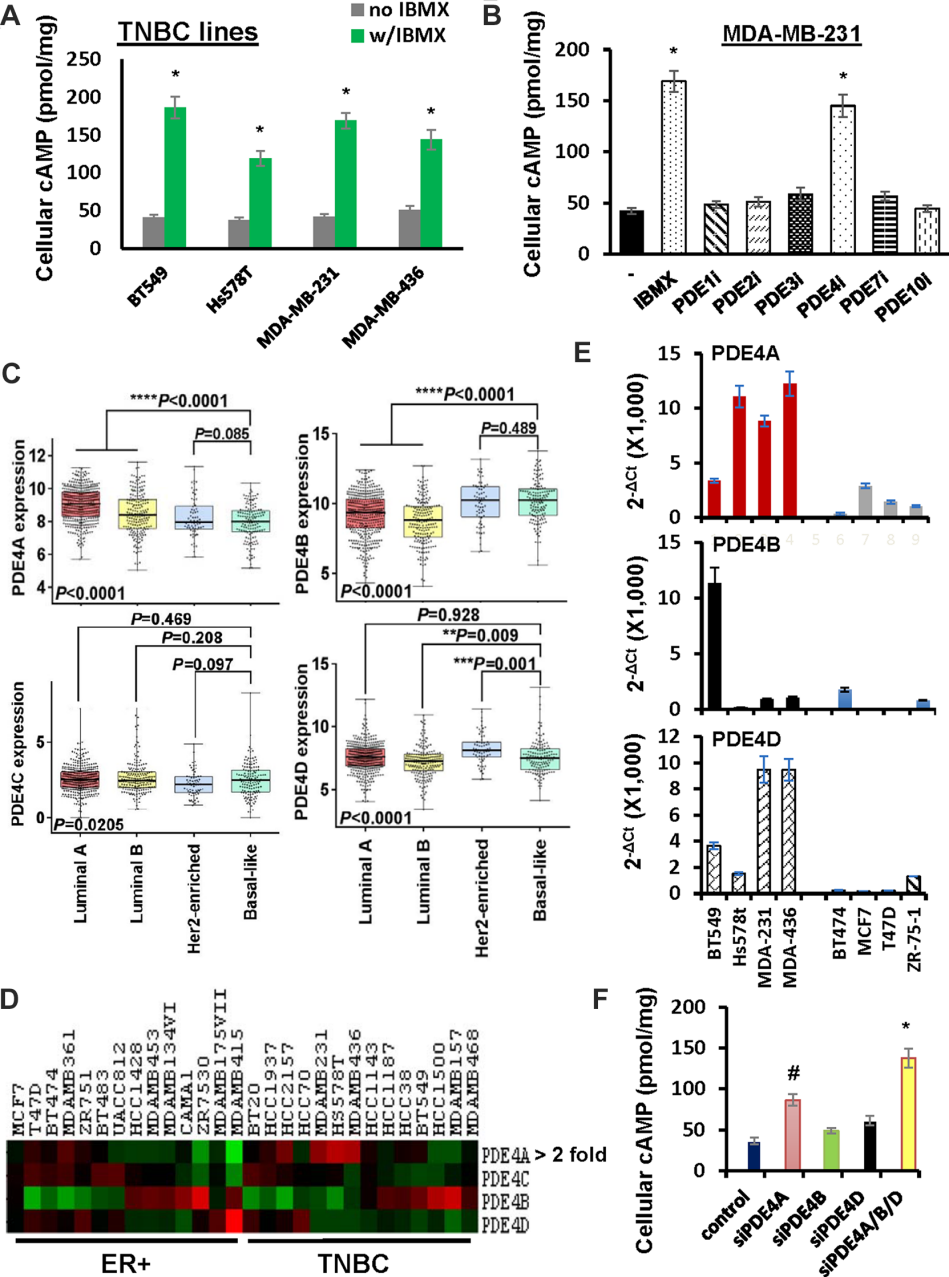
Role of PDE4 isotypes in cAMP decomposition in TNBC cells (**A**) TNBC lines were pretreated with 500 μM probenecid for 1 h followed by treatment of 10 μM forskolin in the absence or presence of 100μM IBMX for 1 h. Cells were collected and analyzed for amount of cAMP. Data are means ± SD (*n* = 3). *P < 0.01 *vs* no IBMX. (**B**) MDA-MB-231 cells were pretreated with 500 μM probenecid for 1 h followed by treatment with 10μM forskolin in the absence or presence of a particular PDE inhibitor. Cells were collected and analyzed for amount of cAMP. Data are means ± SD (*n* = 3). *P < 0.01 *vs* no inhibitor. Vinpocetin (100 μM, PDE1 inhibitor); BAY60-7550 (0.5 μM, PDE2 inhibitor); Cilostazol (3 μM, PDE3 inhibitor); Rolipram (3 μM, PDE4 inhibitor); BRL 50481 (10 μM, PDE7 inhibitor); PF-2545920 (0.5 μM, PDE10 inhibitor). (**C**) Box-and-whisker plot was generate to show the expression of PDE4 isotypes in breast tumor subtypes. (**D**) Heat map was generated to reveal the expression of PDE4 isotypes in established ER+ and TNBC cell lines. (**E**) QRT-PCR of PDE4 isotypes in breast cancer cell lines. Level of β-actin mRNA was used as an internal control for standardization. Data are means ± SD (*n* = 3). (**F**) MDA-MB-231 cells were transfected with control siRNA, PDE4A, PDE4B or PDE4D siRNA pool for 4 days and then pretreated with 500 μM probenecid followed 1-h stimulation of 10 μM forskolin. Cells were collected and analyzed for the amount of cAMP. Data are means ± SD (*n* = 3). *P < 0.005 *vs* control siRNA.

The observation that IBMX reduced the rate of cAMP clearance in TNBC cells (Figure 2C and 2D) implicated a possible role of PDEs in cAMP diminution. To test it, BT549, Hs578T, MDA-MB-231, MDA-MB-436 cells were pretreated with probenecid and then stimulated with forskolin in the absence or presence of IBMX for 1 h followed by analyzing amount of cellular cAMP. Level of cellular cAMP in cells co-treated with forskolin and IBMX was nearly four times higher than that in cells treated with only forskolin (Figure 5A). These results support the notion that PDEs are involved in cAMP clearance in TNBC cells.

There are 11 families of PDEs and 8 of them can degrade cAMP [24]. To identify the particular PDEs mediating cAMP clearance in forskolin-stimulated TNBC cells, probenecid-pretreated MDA-MB-231 cells were stimulated with forskolin in the presence of inhibitors specific for PDE1, 2, 3, 4, 7, 8 or 10 for 1 h. Compared to cells stimulated with only forskolin, level of cellular cAMP was almost tripled in cells co-treated with forskolin and rolipram (PDE4 inhibitor) but not significantly altered by forskolin with any other inhibitors (Figure 5B). Identical results were also obtained with MDA-MB-436 cells (Supplementary Figure S4). These results suggest that PDE4 family members decompose cellular cAMP in forskolin-stimulated TNBC cells.

As there are four isotypes (PDE4A, 4B, 4C and 4D) in PDE4 family [24], we analyzed the expression of PDE4 isotypes in various breast tumor types with TCGA dataset. The expression of PDE4B was significantly higher in basal-like breast tumors compared with other breast tumor types (Figure 5C). Surprisingly, further analysis of established breast cancer cell lines using datasets from Cancer Cell Line Encyclopedia showed that PDE4A, rather than PDE4B was overexpressed in TNBC cell lines compared with ER+ ones (Figure 5D). We also measured the levels of PDE4 isotypes in both TNBC and ER+ breast cancer cell lines by qRT-PCR. While PDE4C was not detectable in any of these lines, we did not observe a clear difference in PDE4B expression between TNBC and ER+ cells (Figure 5E). However, both PDE4A and PDE4D were expressed in a higher level in TNBC lines compared with ER+ lines (Figure 5E). To experimentally verify PDE4 isotypes involved in cAMP diminution, we depleted PDE4A, PDE4B or PDE4D in MDA-MB-231 cells with the aid of respective siRNA pools. Upon the co-treatment of forskolin and probenecid, we observed that silencing the expression of any of them enhanced the level of cellular cAMP in some extent (Figure 5F). Since the abundance of cellular cAMP in cells with depletion of three PDE4 isotypes reached to that observed in rolipram-treated cells (compare Figure 5F with 5B), these results suggest that multiple PDE4 isotypes are involved in the clearance of cellular cAMP in TNBC cells.

### Combined forskolin, probenecid and rolipram treatment suppresses TNBC cell growth

**Figure 6:**
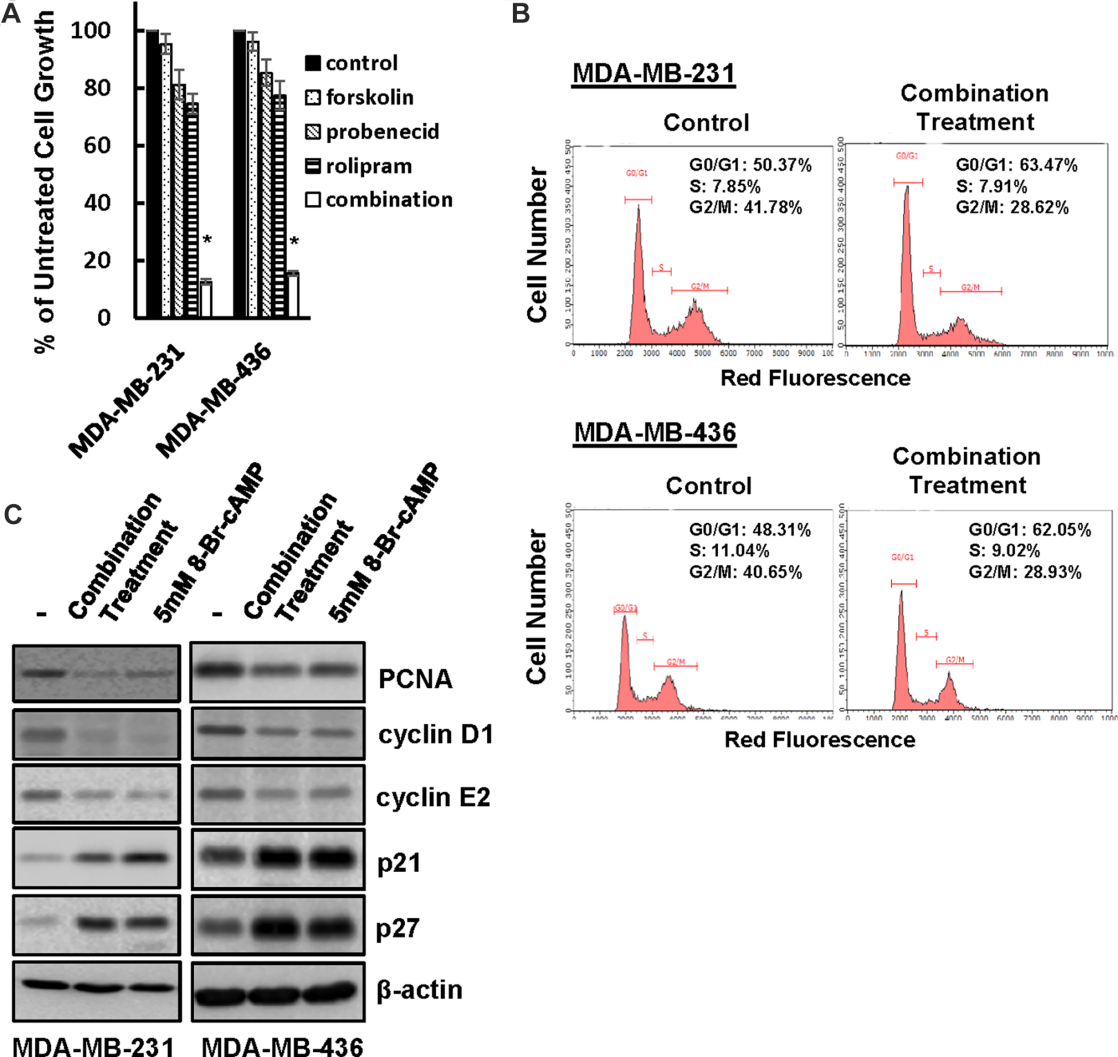
Co-treatment of forskolin, probenecid and rolipram leads to TNBC cell growth arrest (**A**) MDA-MB-231 and MDA-MB-436 cells were treated with 10μM forskolin, 500 μM probenecid and 3 μM rolipram alone or together for 4 days followed by MTT assay to determine cell growth. Data are means ± SD (*n* = 4). *P < 0.001 *vs* control. (**B**) MDA-MB-231 and MDA-MB-436 cells were co-treated with forskolin, probenecid and rolipram or left untreated for 1 day followed by flow cytometry to analyze cell cycle. (**C**) MDA-MB-231 and MDA-MB-436 cells were treated with the combination of forskolin, probenecid and rolipram or 5 mM 8-Br-cAMP or left untreated for 1 day, then lysed and cell lysates were subjected to western blotting to detect cyclin D1, cyclin E2, PCNA, p21, p27 and β-actin with the respective antibodies.

The ability of MRP and PDE4 inhibitors to prevent rapid cellular cAMP diminution in forskolin-stimulated TNBC cells (Figure 5B) provided a window of possibility to suppress TNBC cell growth by combined treatment of forskolin, probenecid and rolipram. To test this possibility, MDA-MB-231 and MDA-MB-436 cells were treated with these agents alone or together for 4 days followed by MTT assay to monitor cell growth. Forskolin displayed little effect on cell growth while probenecid and rolipram were able to slightly inhibit cell growth (Figure 6A). In contrast, treating cells with them together led to over 80% of reduction in cell growth (Figure 6A). These results are consistent with notion that sustaining elevated level of cellular cAMP can lead to the suppression of TNBC cell growth.

To elucidate the molecular mechanism underlying cAMP-induced growth inhibition, MDA-MB-231 and MDA-MB-436 cells were co-treated with forskolin, probenecid and rolipram or left untreated for 1 day followed the analysis of cell cycle progression. Flow cytometry showed that treatment increased population of cells at G0/G1 from approximately 50% to 63% in MDA-MB-231 and 48% to 62% in MDA-MB-436 cells (Figure 6B), indicating a cell cycle arrest at G1 phase. Cell cycle arrest at G1 phase was also consistent with the observation that, similar to 5 mM 8-Br-cAMP, this treatment reduced the abundance of cyclin D1, cyclin E2 and PCNA whereas increased the amount of p21 and p27 (Figure 6C). Since the same treatment did not increase the number of apoptotic cells judging by Annexin/PI-based flow cytometry and PARP cleavage (Supplementary Figure S5), these results suggest that cAMP suppresses TNBC cell growth by arresting cell cycle progression.

### Cocktail of forskolin, probenecid and rolipram suppresses TNBC tumor development

**Figure 7:**
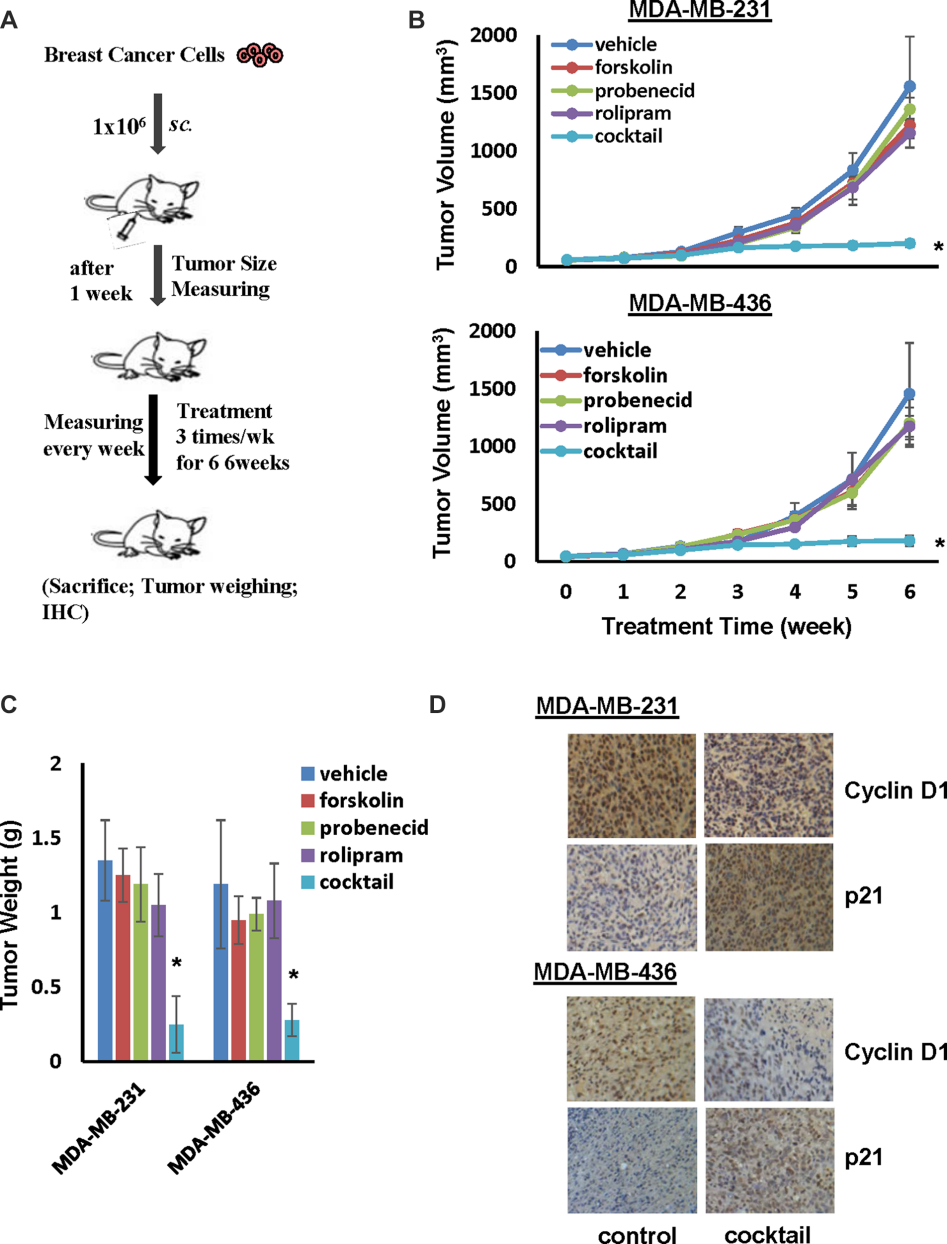
Cocktail of forskolin, probenecid and rolipram suppresses tumor outgrowth of TNBC cells (**A**) Flow chart of therapy scheme. (**B**) MDA-MB-231 or MDA-MB-436 cells (10^6^ cells/mouse) were injected to nude mice for 1 week followed by administering 5mg/kg forskolin, 125 mg/Kg probenecid and 2 mg/ml roliproam alone or together. Tumor development was determined by measuring tumor volume weekly. Data are means ± SD (*n* = 6). *P < 0.01 *vs* vehicle control. (**C**) Weight of tumors excised from mice at the end of treatment. Data are means ± SD (*n* = 6). *P < 0.01 *vs* vehicle control. (**D**) Representative pictures of IHS staining of cyclin D1 and p21 in tumor tissues.

The effectiveness of combined forskolin, probenecid and rolipram treatment to suppress TNBC cell growth led us to investigate the potential of this combination treatment to deter TNBC tumor development with the aid of the well-established orthotopic breast tumor model [25–27]. MDA-MB-231 and MDA-MB-436 cells were injected into mammary fat pad area of female nude mice for 1 week, mice were then randomly divided into 5 groups and each received vehicle, forskolin, probenecid, rolipram or cocktail of all three compounds 3 times a week for 6 weeks (Figure 7A). The dose of forskolin chosen for animal study has previously shown to effectively elevate cellular cAMP level in experimental mouse myeloma model [28] while the doses of probenecid and rolipram was based on their ability to block their respective targets in *in vivo* study [29]. Tumors were evident in all mice at the onset of therapy and progresssed rapidly in mice receiving vehicle (Figure 7B). Administering mice with forskolin, probenecid or rolipram slightly slowed down tumor development (Figure 7B). Strikingly, tumor development almost completely ceased 3 weeks after receiving the cocktail (Figure 7B). At the end of treatment, we weighed tumors collected from sacrificed mice. Compared to control mice, we observed approximately 80% reduction in tumor weight in mice that received cocktail (Figure 7C). In a parallel experiment, we performed identical experiment with ER+ MCF7 cells. While tumor outgrowth was slower with MCF7 cells in comparison with MDA-MB-231 or MDA-MB-436 cells, we only detected moderately suppressive effect in tumor development or final tumor weight in mice receiving cocktail (Supplementary Figure S6A and S6B). These results suggest that cocktail of forskolin, probenecid and rolipram specifically suppress TNBC tumor development.

To link ceased tumor outgrowth to arrested cell cycle progression, we performed immunohistochemistry to examine the intensity of cyclin D1 and p21 staining on collected tumors. Strong cyclin D1 staining was detected in tumors derived from control mice whereas staining of p21 was very weak (Figure 7D). In contrast, tumors derived from mice administered with cocktail displayed little cyclin D1 but robust p21 staining (Figure 7D). These results further support the notion that the stoppage of tumor outgrowth in mice receiving cocktail is the consequence of TNBC cell cycle arrest.

## DISCUSSION

Breast cancer can be classified into four main types on the basis of the presence/absence of several receptors: ER+, PR+, HER2-enriched and TNBC. Hormone therapy is effective for the treatment of most ER+/PR+ breast cancer while HER2-targeted therapy is successfully applied to HER2-enriched breast cancer [30, 31]. However, TNBC-targeted therapy is not available and cytotoxic chemotherapy is the only approved therapy for TNBC. Unfortunately, TNBC patients often develop drug resistance and demise with subsequent metastasis [1]. Since cAMP-elevating agents have been shown to effectively suppress growth of various cancer cell types [7–9], we explored the potential of targeting TNBC with such agents. In this study, we showed that high concentration of cAMP analog 8-Br-cAMP (> 1 mM) specifically suppressed TNBC cell growth (Figure 1A). This finding is in agreement with an early report in which 8-Br-cAMP was shown to inhibit growth of TNBC MDA-MB-231 cells at a concentration above 1mM [21]. Since it is infeasible to use almost any agent at such high dose in clinic, we tested the potential of adenylate cyclase activator forskolin and PDE inhibitor IBMX as an alternative. Unfortunately, neither alone or together exhibited significant inhibitory effect on TNBC cell growth (Figure 1B).

Adenylate cyclase-mediated production and PDE-mediated degradation are thought to be the mechanism controlling the level of cellular cAMP. We revealed that forskolin quickly induced production of cAMP in both TNBC and ER+ breast cancer cells (Figure 2A). However, a rapid diminution of cellular cAMP followed the initial cAMP production in TNBC but not ER+ cells. The rapid clearance of cellular cAMP was obviously not due to PDE-mediated decomposition because pan-PDE inhibitor IBMX only slightly slowed the rate of cellular cAMP diminution (Figure 2). Instead, we revealed that MRP1/4-mediated efflux was a principal mechanism eradicating cellular cAMP while PDE4A was also able to decompose cAMP when MRPs were blocked (Figures 3, 4 and 5). Our results thus add MRP-mediated efflux as a principal mechanism TNBC cells employ to control cellular cAMP level.

Early studies have reported that agents raising cAMP are able to suppress growth of colon and medullary thyroid cancer cells [32, 33]. However, we found that commonly-used cAMP-elevating agents exhibited little effect on TNBC cell growth (Figure 1). We reason that inability of cAMP-elevating agent to deter TNBC cell growth was due to the coordinated action of rapid MRP1/4-mediated cAMP efflux and PDE4-mediated cAMP decomposition. This is apparently the case because forskolin is only able to significantly curb TNBC cell growth in the presence of MRP and PDE4 inhibitors (Figure 6). Similarly, we found that only the cocktail of forskolin, probenecid and rolipram was able to cease TNBC tumor outgrowth (Figure 7). Early studies indicate that cAMP might inhibit cell growth by inducing cell cycle arrest or apoptosis [7, 9, 34]. Our results pinpoint the former as the mechanism because combined treatment of forskolin, probenecid and rolipram arrested cell cycle at G1 phase but did not induce apoptosis (Figure 6 and Supplementary Figure S5). Moreover, this treatment also decreased levels of cyclin D1/E2 while increased abundance of p21 and p27 in TNBC cells (Figure 6).

Our results showed that only growth of TNBC but not ER+ breast cancer cells was sensitive to elevated level of cellular cAMP (Figures 1, 6 and 7). TNBC cells’ selective sensitivity to elevated level of cAMP may explain why cAMP is rapidly diminished in these cells but not in ER+ cells (Figure 2). As knockdown of MRP1/4 largely prevented rapid efflux of cellular cAMP (Figure 4) while depletion of PDE4A/B/D blocked cAMP decomposition, we conclude that MRP1/4 and multiple PDE4 isotypes work in concert to diminish cAMP in TNBC cells. MRP1 and 4 are overexpressed in basal-like breast tumors and established TNBC cell lines (Figure 3). This observation is consistent with their role to facilitate cAMP diminution in TNBC cells. Our findings suggested the possibility of targeting TNBC by raising the level of cellular cAMP through interfering with both MRP1/4 and PDE4 functions. This possibility is evidently supported by our observation that cocktail of forskolin, probenecid and rolipram at clinically reasonable doses suppressed TNBC, but not ER+ breast tumor outgrowth (Figure 7). As inhibitors specific for MRP and PDE4 are approved for clinic use, our study has thus laid a foundation for a novel TNBC-targeted therapy with these compounds.

## MATERIALS AND METHODS

### Cell culture and reagents

All cell lines were purchased from American Tissue Culture Collection within the past 6 months and cultured in DMEM supplemented with 10% fetal bovine serum. Information on all agonists and inhibitors is provided in Supplemental Materials. Control siRNA, MRP and PDE4 siRNA pools were purchased from GE Dharmacon (Lafayette, CO).

### MTT and clonogenic assays

3-(4,5-dimethylthiazol-2-yl)-2,5-diphenyltetrazolium bromide (MTT) assay was performed to analyze cell growth as previously described [35–37]. In each assay, 5x10^3^ cells were seeded into each well of 24-well culture plates for overnight followed by treatment of 8-Br-cAMP or other agents for 4 days. Clonogenic assay was performed to determine the inhibitory effect of high concentration of 8-Br-cAMP. Briefly, 5 × 10^3^ cells were seeded in 6-cm dishes in the absence or presence of 1 mM 8-Br-cAMP for 7 days and media were replaced once every other day. Cells were fixed with 0.5% glutaraldehyde and then stained with 0.05% crystal violet. Colonies were counted under a dissecting scope.

### Measurement of cAMP

Amount of cAMP was analyzed using Cyclic AMP Assay Kit (Cell Signaling Technology, Danvers, MA). Briefly, overnight-cultured cells were pretreated with PDE inhibitors, probenecid or other inhibitors for 1 h and then stimulated with forskolin for varying length of times. Both cells and media were collected for cAMP measurement. cAMPs in cells and media were considered as cellular and secreted cAMP respectively. To investigate the importance of MRPs or PDE4s, cells were transfected with control siRNA or siRNA pools for distinct MRP or PDE isotype for 4 days prior to the analysis of cAMP.

### Flow cytometry to analyze cell cycle and apoptosis

To analyze the status of cell cycle, cells were detached with trypsin, washed and fixed in 100% ethanol. After a brief centrifugation to remove ethanol, cells were suspended in PBS containing 20 μg/mL of propidium iodide followed by flow cytometry analysis using FACSCanto II flow cytometer (BD Biosciences, Bedford, MA). The data were analyzed using the BD FACSDiva Software.

### Western blotting

Western blotting was performed as previously described [37]. Information on all antibodies is provided in Supplemental Materials. Level of β-actin was determined for every blot as an internal loading standard.

### *In vivo* studies

All procedures were conducted with animal welfare considerations and approved by the Ethical Committee of Shanghai University of Traditional Chinese Medicine and performed as previously described [25–27]. Briefly, 1 × 10^6^ cells were injected into 4th mammary fat pad of 6-week-old female BALB/c nude mice (Academia Sinica, Shanghai, China). After 1 week, mice were randomly divided into five groups (6 mice/group) and received treatment 3 times a week for 6 weeks. Five groups were vehicle (control), forskolin, probenecid, rolipram, and cocktail of forskolin, probenecid and rolipram. Tumor development was monitored by weekly measuring tumor volumes (V) which were calculated using formula of V = 0.5 x Dmax x (Dmin)^2^, where Dmax is the maximal tumor diameter and Dmin is the corresponding perpendicular diameter.

### Statistical analysis and bioinformatics

Statistical differences were calculated using 2-tailed Student *t* test. The expression of MRP family members and PDE4 isotypes in various breast tumor types was evaluated using dataset TCGA_BRCA_exp_HiSeqV2 (https://genome-cancer.ucsc.edu) and shown in box-and-whisker plots. Interquartile range (IQR) was expressed by the colored box and the bar indicated the median value. Statistical difference was calculated using Analysis of variance. To compare the expression of MRP family members and PDE4 isotypes between TNBC and ER+ breast cancer cell lines, expression data of various breast cancer cell lines from Cancer Cell Line Encyclopedia (http://www.broadinstitute.org/ccle/home) were retrieved. CCLE_Expression_2012-10-18.res and CCLE_Expressoin_2012-09-29.res datasets were respectively used to evaluate the expression of MRP family members and PDE4 isotypes. Heat maps (green-black-red, representing low-medium-high expression respectively) were constructed by Gene Cluster 3.0.

## SUPPLEMENTARY FIGURES


