# Supplementary material for: Triple negative breast cancer development can be selectively suppressed by sustaining an elevated level of cellular cyclic AMP through simultaneously blocking its efflux and decomposition
Source: Oncotarget. 2016 Nov 25;7(52):87232–45. doi: 10.18632/oncotarget.13601 (PMC5349984; doi:10.18632/oncotarget.13601)
Supplement: Supplementary file 1 [file oncotarget-07-87232-s001.pdf]

Triple negative breast cancer development can be selectively suppressed by sustaining an elevated level of cellular cyclic AMP through simultaneously blocking its efflux and decomposition

Supplementary Materials

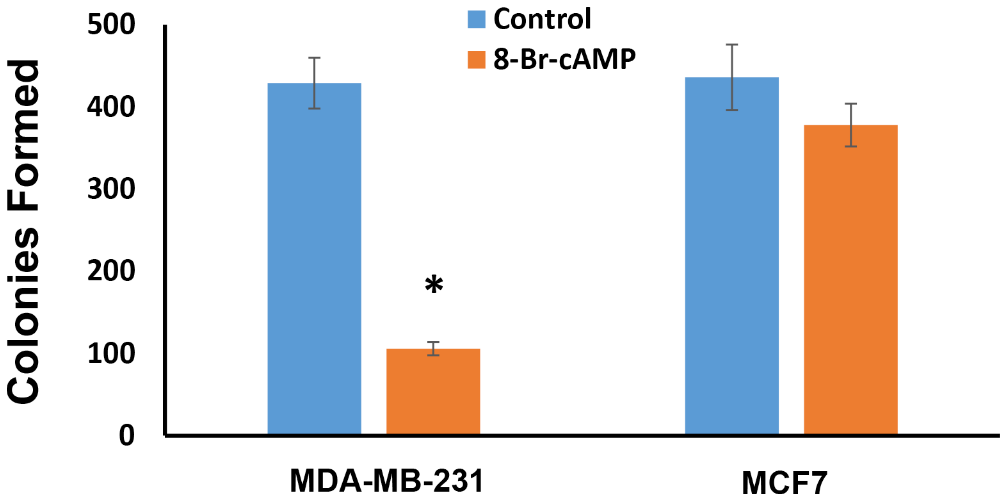

Supplementary Figure S1: Effect of high concentration of 8-Br-cAMP on clonogenic abilities of TNBC and ER+ breast cancer cells. MDA-MB-231 or MCF7 cells (5×10^3 cells/dish) were seeded in 6-cm dishes in the absence or presence of 1 mM 8-Br-cAMP for 7 days and media were replaced once every other day. Cells were fixed with 0.5% glutaraldehyde and then stained with 0.05% crystal violet. Colonies were counted under a dissecting scope. Data are means ± SD (n = 3). *$P < 0.001$ vs no 8-Br-cAMP.

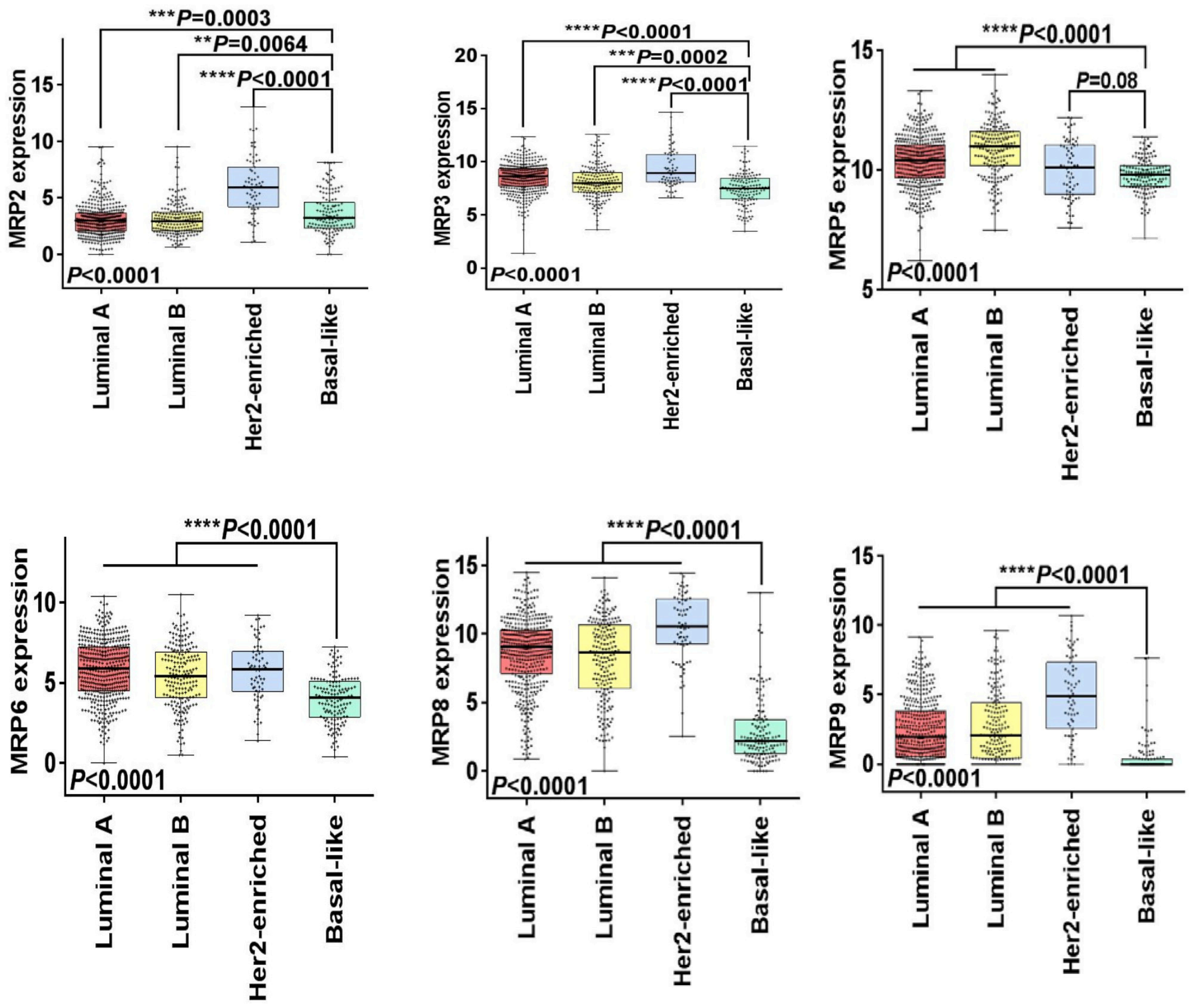

Supplementary Figure S2: Association of various members of MRP family with breast tumor subtypes. Box-and-whisker plot was generated using dataset from TCGA_BRCA_exp_HiSeqV2 data set. *P* values were calculated with ANOVA.

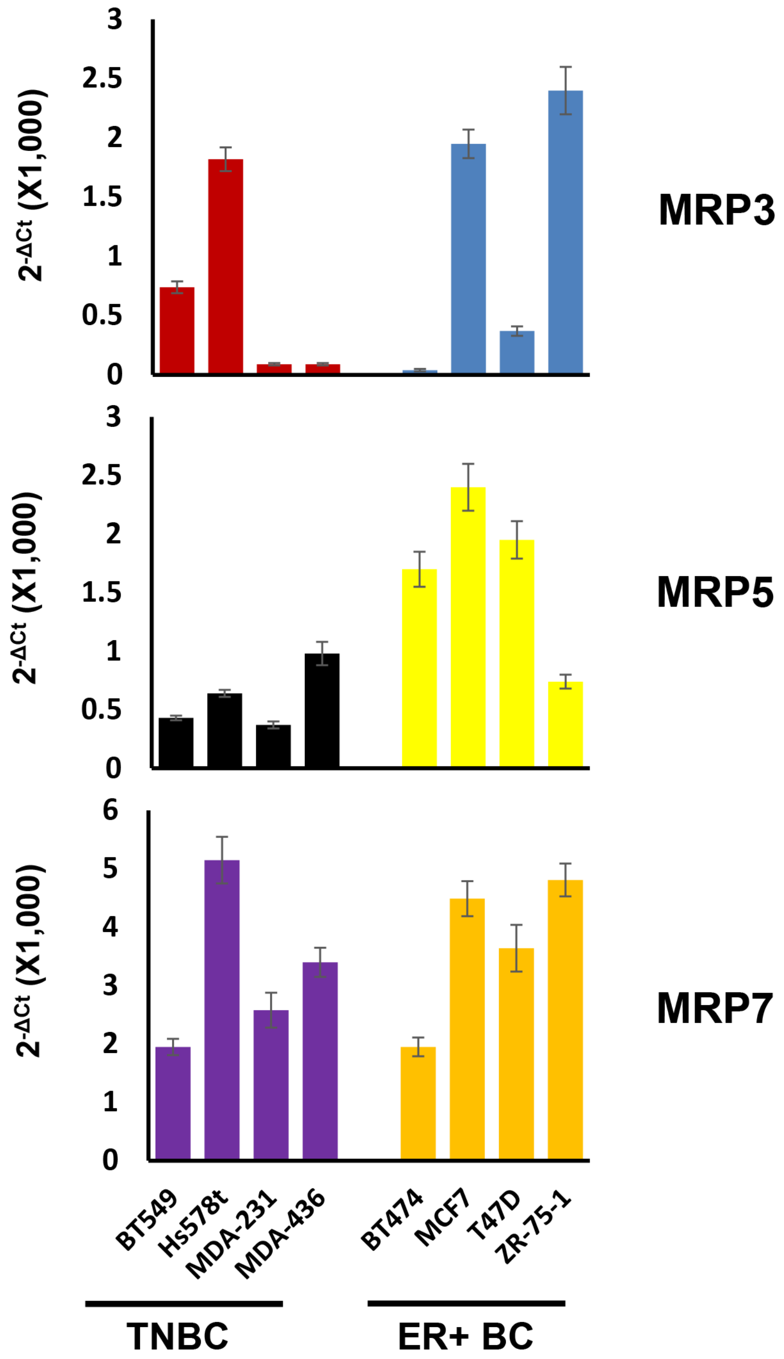

Supplementary Figure S3: QRT-PCR of MRP3, 5 and 7 in TNBC and ER+ breast cancer cell lines. Total RNA was isolated from overnight-cultured cells and used for examine the levels of MRP3, 5 and 7 mRNA. Level of β-actin mRNA was also measured and used as an internal control for standardization. Data are means ± SD (n = 3).

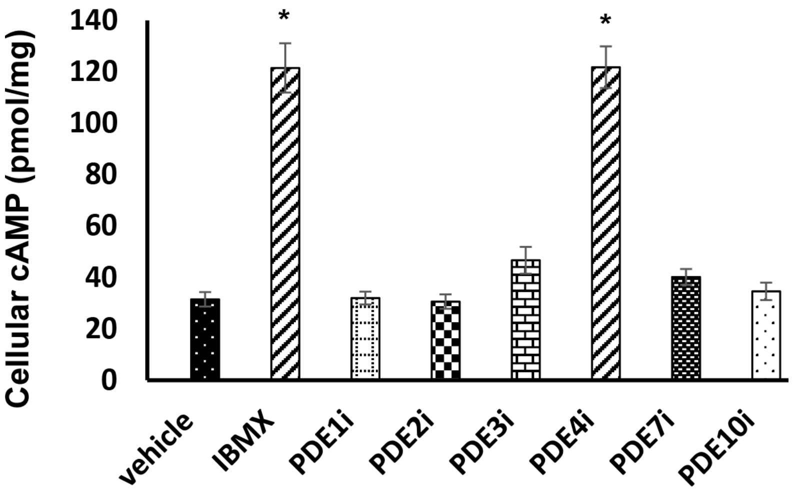

Supplementary Figure S4: PDE4 contributes to rapid diminution of cellular cAMP in TNBC cells. MDA-MB-436 cells were pretreated with pretreated with 1 mM probenecid for 1 h followed by treatment with 10 µM forskolin in the absence or in presence of a particular PDE inhibitor. IBMX–100 µM, pan-PDE inhibitor; vinpocetin–100 µM, PDE1 inhibitor; BAY60-7550–0.5 µM, PDE2 inhibitor; cilostazol–3 µM, PDE3 inhibitor; Rolipram–3 µM, PDE4 inhibitor; BRL 50481–10 µM, PDE7 inhibitor; PF-2545920–0.5 µM, PDE10 inhibitor.

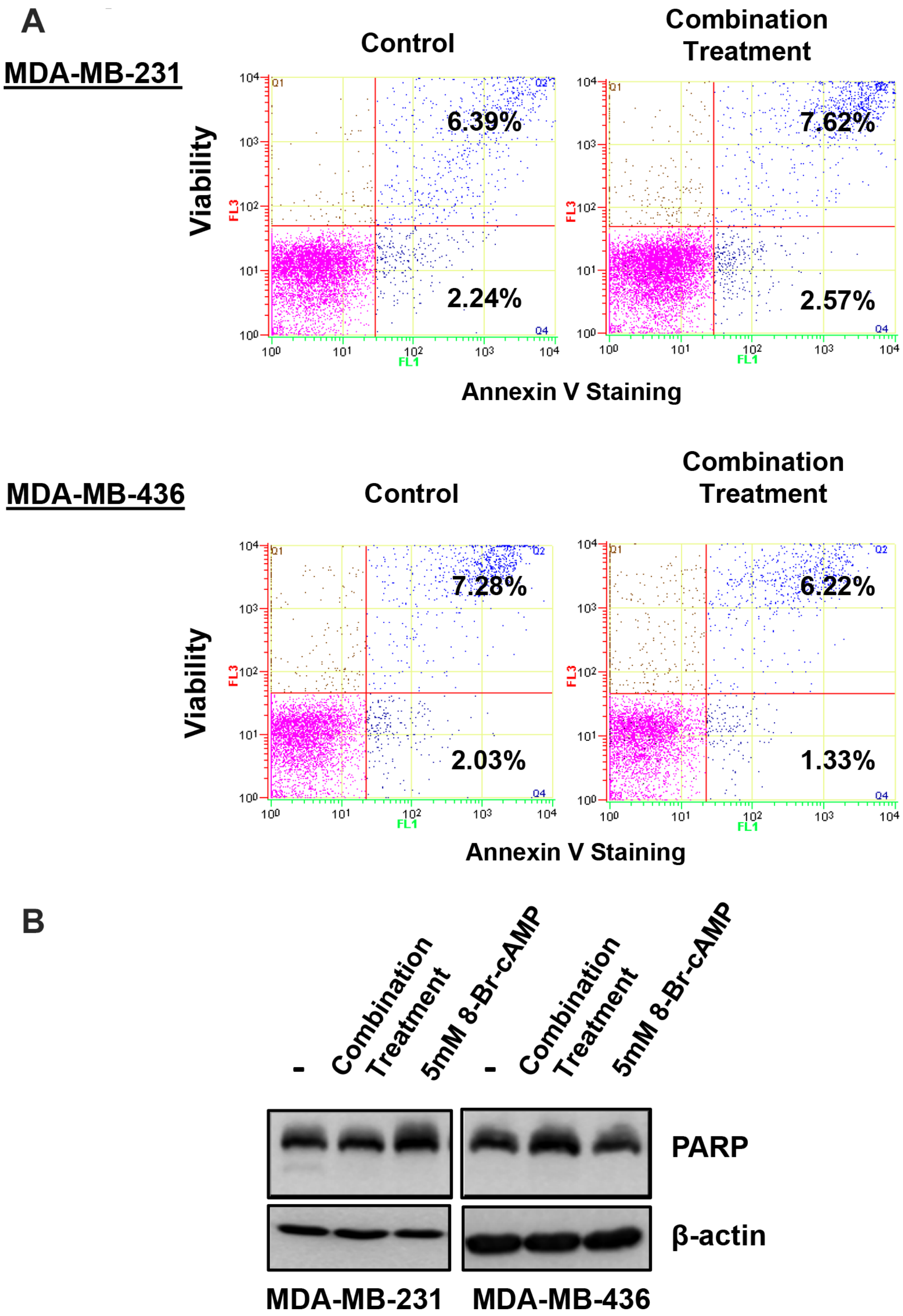

Supplementary Figure S5: Sustaining an elevated level of cellular cAMP does not induce apoptosis. (**A**) MDA-MB-231 and MDA-MB-436 cells were treated with the combination of 10 µM forskolin, 500 µM probenecid and 3 µM rolipram or left untreated for 1 day and subjected to Anexin V-based flow cytometry to analyze cell apoptosis. (**B**) Cells were treated with the combination of forskolin, probenecid and rolipram or left untreated for 1 day, then lysed for Western blotting to detect PARP with PARP polyclonal antibody.

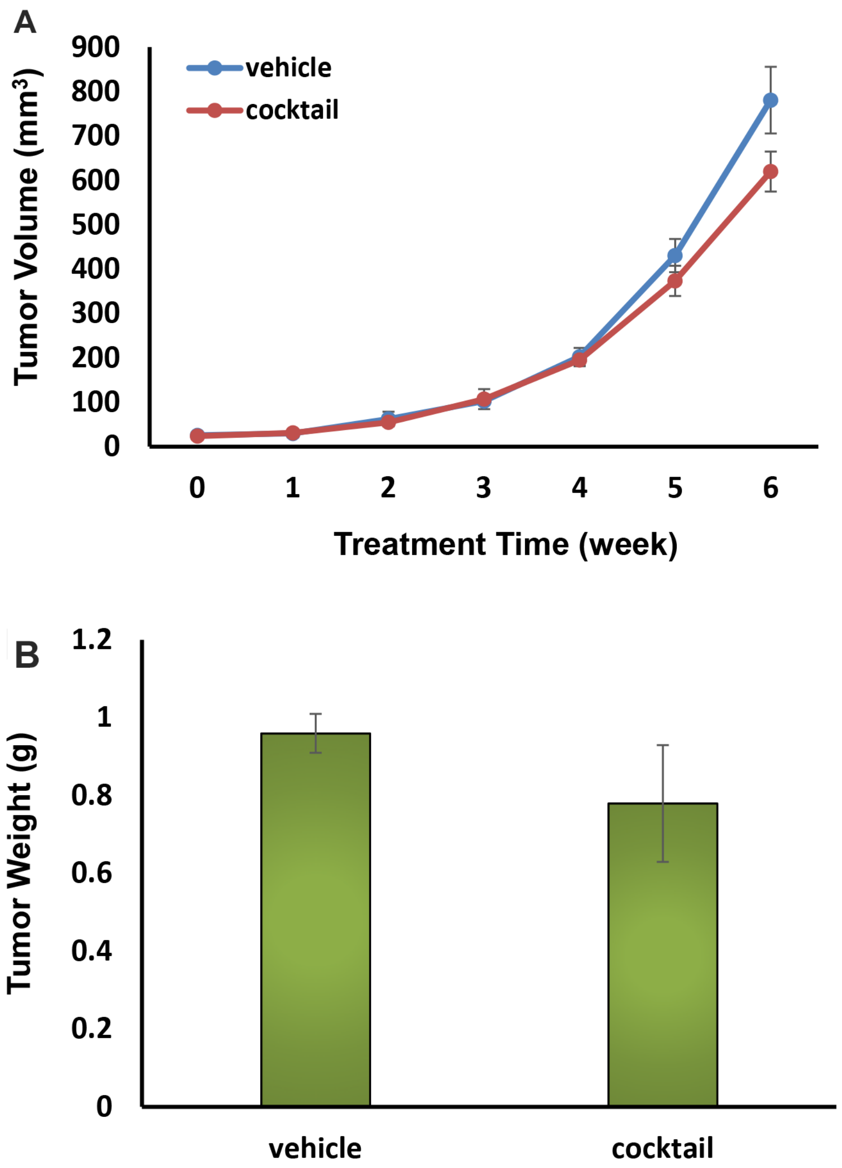

Supplementary Figure S6: Effect of forskolin, probebecid and rolipram cocktail on tumor development of MCF7 cells. (**A**) MCF7 cells (10^6 cells/mouse) were injected to nude mice for 1 week followed by administering 5 mg/kg forskolin, 125 mg/Kg probenecid and 2 mg/ml roliproam alone or together. Tumor development was determined by measuring tumor volume weekly. Data are means ± SD (*n* = 6). (**B**) Weight of tumors excised from mice at the end of treatment. Data are means ± SD (*n* = 6).
